# An Injection Molded SlipChip with Self‐Sampling for Integrated Point‐of‐Care Testing of Human Papilloma Virus

**DOI:** 10.1002/advs.202406367

**Published:** 2024-09-25

**Authors:** Jiajie Zhang, Zhangli Dong, Lei Xu, Xu Han, Zheyi Sheng, Weiyu Chen, Jiayi Zheng, Dongmei Lai, Feng Shen

**Affiliations:** ^1^ School of Biomedical Engineering Shanghai Jiao Tong University 1954 Huashan Road Shanghai 200030 China; ^2^ The International Peace Maternity and Child Health Hospital, School of Medicine Shanghai Jiao Tong University Shanghai 200030 China; ^3^ Shanghai Key Laboratory of Embryo Original Diseases Shanghai 200030 China; ^4^ Hefei Early Cancer Screening Innovation Technology Institute Hefei Inovation Industrial Park Wangjiang West Road Hefei China

**Keywords:** HPV screening, lab on a Chip, microfluidics, point of care, self‐sampling, SlipChip

## Abstract

High‐risk human papillomavirus (HPV) screening is crucial for cervical cancer prevention. However, laboratory‐based nucleic acid amplification tests (NAATs) require costly equipment, designated lab space, and skilled personnel. Additionally, cervical swabs collected by healthcare professionals can be inconvenient, uncomfortable, and reduce privacy, limiting broader application and patient compliance. A SlipChip‐based Integrated Point‐of‐Care (SIPOC) system featuring an injection‐molded SlipChip is presented with preloaded reagents for nucleic acid extraction and a portable four‐channel real‐time quantitative PCR instrument for detection. This system incorporates a self‐sampling method that allows participants to collect their own vaginal swabs, with the β‐Globin gene as a control. After testing 130 participants for HPV‐16 and HPV‐18, 97.7% of the self‐collected samples are valid. Among valid samples, 25 tested positive for HPV‐16 and 9 for HPV‐18. Compared to Roche's standard HPV PCR test, the SIPOC system shows 100% positive predictive value (PPV) for both HPV‐16 and HPV‐18 and negative predictive values (NPVs) of 99.0% and 99.1%, respectively. This system is promising for HPV screening in resource‐limited settings and adaptable for other point‐of‐care NAAT applications, including home testing.

## Graphical abstract

High‐risk HPV screening is essential for cervical cancer prevention. This study develops an injection‐molded SlipChip‐based Integrated Point‐of‐Care system that simplifies the HPV screening process with self‐collected samples, a series of slipping operations, and a portable four‐channel quantitative PCR instrument. This user‐friendly method offers a promising platform for HPV screening, particularly in resource‐limited settings and even for home testing.

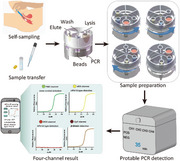

## 1 Introduction

The human papillomavirus (HPV) is a sexually transmitted virus that affects a significant portion of the global population.^[^ ^1^, ^2^ ^]^ Persistent infection with high‐risk HPV subtypes, such as HPV‐16 and HPV‐18, significantly increases the risk of cervical cancer.^[^ ^3^, ^4^, ^5^ ^]^ Although vaccines for HPV are available, their adoption is limited due to factors such as restricted availability and specific age recommendations, typically ranging from 9 to 14 years.^[^ ^6^, ^7^, ^8^ ^]^ Consequently, regular screening for high‐risk HPV is critical for controlling and eventually eliminating cervical cancer.^[^ ^9^, ^10^ ^]^ The World Health Organization (WHO) recommends that 70% of women undergo high‐quality screening by ages 35 and 45.^[^ ^11^ ^]^ Regular HPV screening is also advised for adult women beyond these age groups.^[^ ^12^, ^13^ ^]^ However, current screening rates, especially in developing and resource‐limited regions, fall significantly below this goal, primarily due to limited test availability, high costs, and privacy concerns.^[^ ^14^, ^15^, ^16^ ^]^

A variety of methods have been developed for HPV screening.^[^ ^17^, ^18^, ^19^ ^]^ The most common method for HPV identification involves nucleic acid amplification tests (NAATs), such as polymerase chain reaction (PCR).^[^ ^20^, ^21^, ^22^ ^]^ PCR‐based methods offer high sensitivity and specificity, with many commercialized assays demonstrating significant clinical value in HPV diagnosis. However, PCR tests are time‐consuming, require advanced equipment and laboratory space, and depend on trained personnel, limiting their broader application, especially at point‐of‐care and in resource‐limited settings.^[^ ^23^, ^24^, ^25^ ^]^ To address these challenges, alternative HPV testing methods have emerged, including isothermal amplification techniques,^[^ ^26^, ^27^ ^]^ lateral flow assays (LFA),^[^ ^28^ ^]^ reverse dot blots,^[^ ^29^ ^]^ and CRISPR‐Cas‐based analysis methods.^[^ ^30^, ^31^ ^]^ Microfluidic systems, often called “Lab on a Chip,” show potential for point‐of‐care testing.^[^ ^32^, ^33^ ^]^ However, these innovations require further refinement in terms of scalability, precision, and robustness, preventing them from replacing central laboratory‐based PCR tests for HPV.

Additionally, the method of sample collection is another key factor influencing test accessibility and individuals' willingness to undergo the procedure. Currently, cervical swab samples are typically collected by healthcare professionals in hospitals or laboratories, often using cervical forceps, causing discomfort and privacy concerns.^[^ ^34^ ^]^ Self‐sampling could be a more acceptable alternative, allowing individuals to collect their own samples from the vaginal area.^[^ ^35^, ^36^, ^37^ ^]^ However, self‐sampling raises concerns about potential errors, such as false negatives, and whether self‐collected samples provide comparable diagnostic results to those obtained by professionals.^[^ ^38^, ^39^ ^]^ Further studies are needed to validate the accuracy and reliability of self‐sampling in HPV testing.

In this work, we developed a SlipChip‐based integrated point‐of‐care (SIPOC) system with self‐sampling for high‐risk HPV detection. This SIPOC system comprises an injection‐molded SlipChip microfluidic device that can be manufactured on a large scale and at a relatively low cost, along with a portable four‐color real‐time quantitative PCR instrument. The SIPOC system streamlines the current laboratory‐based HPV testing protocol by integrating magnetic bead‐based nucleic acid sample preparation with PCR amplification and detection. The process begins with a self‐collected vaginal swab, from which the eluted sample is transferred to the SlipChip device. Through a series of simple sliding operations, the system accomplishes viral lysis, nucleic acid extraction, and purification. Following these steps, the system performs real‐time PCR to simultaneously analyze HPV‐16, HPV‐18, β‐Globin, and an external control using the portable four‐color real‐time q‐PCR system. This approach offers a more accessible, efficient, and user‐friendly method for high‐risk HPV detection in point‐of‐care settings.

To demonstrate the clinical utility of the SIPOC system, 130 participants self‐collected vaginal swab samples, which were then tested using the SIPOC system. Meanwhile, standard sample collection and analysis were performed using the Roche Cobas system as a control. Our results showed that 97.7% of the self‐collected samples detected the β‐Globin gene, indicating that nearly all participants successfully completed the self‐sampling process. The system demonstrated a positive predictive value (PPV) of 100% for both HPV‐16 and HPV‐18 and a negative predictive value (NPV) of 99.0% for HPV‐16 and 99.1% for HPV‐18. The SIPOC system not only provides accurate detection of HPV‐16 and HPV‐18 but also offers relative quantification data, such as viral load, through normalization with an external standard. With further modifications, verifications, and validations, the SIPOC system could be adapted for testing a variety of diseases in resource‐limited settings and potentially for home use.

## 2 Results

**Figure 1:**
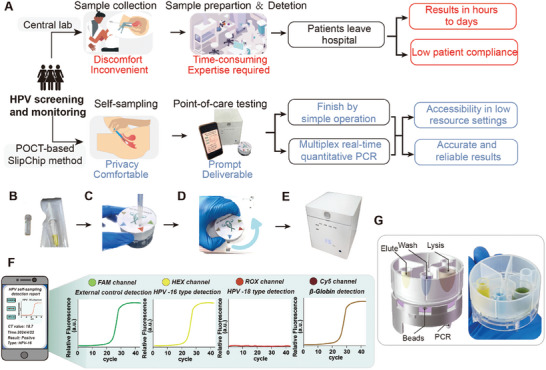
SIPOC platform design and operation. A) Comparison of standard‐of‐care for HPV screening and monitoring in centralized laboratories (top) versus the home‐based SlipChip method with the SIPOC platform (bottom). B–F) Photographs illustrating the SIPOC platform and self‐sampling kit: B) The self‐sampling kit. C) Transferring the sample. D) Sample preparation using the SlipChip. E) Four‐color real‐time quantitative PCR reaction. F) Schematic showing the system interfacing with a smartphone or tablet via Bluetooth for real‐time monitoring of PCR amplification. PCR is used to determine whether HPV‐16/18 is present or if the sample collection is valid. HPV‐16 is detected in the HEX channel, HPV‐18 in the ROX channel, the β‐Globin gene in the CY5 channel, and the external control in the FAM channel to confirm reagent usability. G) Schematic and photograph depicting the sample preparation process using the SlipChip after sample transfer. The steps include lysis and binding, washing, and elution with preloaded buffers, achieved by rotating and shaking the chip to move the magnetic beads.

To conduct HPV screening and NAATs in resource‐limited settings, we developed a convenient and user‐friendly process using the SIPOC platform with self‐sampling (**Figure** **1**A). Traditional HPV testing typically requires patients to queue at a hospital for cervical swabbing, usually conducted by a physician using cervical forceps, which involves applying external force to dilate the vaginal canal, often causing discomfort and bleeding. After collection, the sample is sent to a centralized laboratory for nucleic acid extraction and amplification, using specialized equipment like the Roche Cobas 4800. The entire process is inconvenient, contributing to lower patient compliance due to its invasive nature and long wait times for test results. The reliance on specialized equipment and trained personnel also limits access to HPV DNA testing in resource‐poor regions, potentially delaying cervical cancer diagnosis.

Using the current SIPOC platform for HPV detection involves three steps. First, users collect a vaginal swab sample using a self‐sampling device and transfer the sample (Figure 1B,C). Next, the sample is prepared using the injection‐molded SlipChip (Figure 1D). Finally, four‐channel PCR quantitative detection is performed. HPV detection utilizes an integrated four‐color fluorescence detector with probes targeting HPV‐16, HPV‐18, β‐Globin, and an external control (Figure 1E). Results are wirelessly transferred to a mobile phone for analysis and display (Figure 1F), with synchronization to a central database for remote diagnosis. All nucleic acid sample preparation reagents and lyophilized amplification reagents are pre‐stored within the device (Figure 1G). The entire process, from sample collection to results, takes <60 min, significantly reducing the time required for HPV nucleic acid testing and enhancing ease of use.

**Figure 2:**
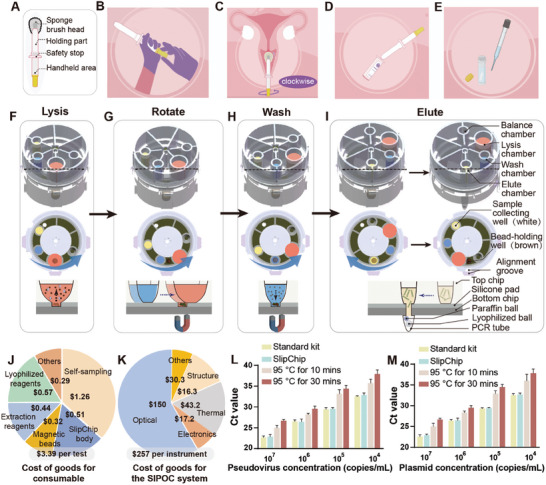
Workflow for sample preparation and SlipChip characterization. A–E) The workflow of self‐sampling: A) Structure of the self‐sampler. B) Preparing for sampling. C) Inserting the sampler into the vagina to collect the sample. D) Sample collected into the preservation buffer. E) Transferring the sample into the SlipChip. F–I) Diagram of the sample preparation workflow for the SlipChip: The red reagent represents the lysis buffer and the sample. The solid brown circles represent magnetic beads. The blue reagent represents the washing buffer. The yellow reagent represents the elution buffer. The blue ball in the centrifuge tubes represents paraffin wax. The white ball in the centrifuge tube represents the lyophilized reagent for the PCR reaction. The brown circle in the top view represents the bead‐holding well. The white circle in the top view represents the sample collecting well. The blue arrow shows the direction of the chip at each step. F) The initial position of the SlipChip after sample transfer, indicating the shaking step. G) The rotation step to align the bead‐holding wells with the washing chamber. H) The step for magnetic bead pelleting. I) The elution and sample collection step. J) Cost of goods for consumable. K) Cost of goods for the SIPOC system. L) Bar chart presenting the threshold cycle (Ct) values from q‐PCR for samples containing serially diluted HPV‐16 pseudoviruses after extraction by the standard kit, SlipChip, 95 °C heating for 10 min, and 95 °C heating for 30 min. The error bars represent the standard deviations (n = 3). M) Bar chart presenting the Ct values from q‐PCR for samples containing serially diluted HPV‐16 plasmid DNA after extraction by the standard kit, SlipChip, 95 °C heating for 10 min, and 95 °C heating for 30 min. The error bars represent the standard deviations (n = 3).

We introduced self‐sampling for vaginal swabs to improve convenience, comfort, and privacy. Participants were instructed to collect their own vaginal swabs, while healthcare professionals collected a parallel sample using standard procedures (supplemental methods). All 130 participants successfully followed the self‐sampling protocol (**Figure** **2**A–E), and feedback indicated that the experience was better compared to traditional methods. The design of the self‐sampling device is shown in Figure 2A, and the self‐sampling process is described in Supporting Information.

We developed an injection‐molded SlipChip for nucleic acid sample preparation, designed to operate with a series of slipping processes (Figures 2F–I). The upper chip is constructed from polycarbonate (PC) material, and the lower chip is constructed from polypropylene (PP) material (Figure S1, Supporting Information). All necessary reagents for sample preparation are preloaded and sealed within the SlipChip device. During operation, the sample moves through different chambers for lysis, washing, elution, and ultimately, collection in the sample tube. The entire sample preparation process has three key stages: magnetic bead resuspension through shaking, magnetic bead pelleting, and chip rotation for chamber alignment.

Users were given the following list of instructions: First, the entire chip is shaken to resuspend the magnetic beads, enabling viral lysis and nucleic acid capture by the beads (Figure 2F). Similarly, for the washing and elution steps, the chip is thoroughly shaken to ensure proper mixing. After each shaking step, the chip is placed on the base of the SIPOC system, where a magnet settles the magnetic beads within 10 s (Figure 2H). To transition from the lysis step to the washing step, the upper chip is rotated to align the bead‐holding wells with the washing chamber while on the base of the SIPOC system, where the magnet secures the magnetic beads in place (Figure 2G). This same rotation method is used to switch from the washing step to the elution step, and then from the elution step to the sample collection step. After completing the elution step, the upper chip is rotated counterclockwise to align the bead‐holding wells with the elution chamber, ensuring the sample collection tube is in position. The chip is shaken to transfer the liquid and dissolve the lyophilized reagents, thereby capturing the nucleic acids (Figure 2I). This process efficiently manages the sequential transitions required for successful nucleic acid extraction and collection, leveraging rotational alignment and magnetic bead control. The purified nucleic acids are collected in the amplification tube attached to the device. The β‐Globin gene serves as a critical internal control in the assay, ensuring proper sample collection and confirming the presence of human cells. Although not specific to HPV, it is a human gene expressed in red blood cells.^[^ ^40^ ^]^ Its stable expression and highly conserved sequence make it an ideal control to ensure that the collected sample contains sufficient human cellular material for reliable testing. Additionally, the external control verifies the performance of the amplification reagents, providing an extra layer of validation for the assay.

By using injection molding, we have significantly reduced the manufacturing cost of the entire chip, lowering the cost of goods for the consumable to less than $3.40 (Figure 2J). Additional characterization details of the injection‐molded SlipChip are provided in the supporting information. We also assessed the current cost of goods for the SIPOC system (Figure 2K; Table S2, Supporting Information) and anticipate further cost reductions with large‐scale production.

To evaluate the extraction performance of the SlipChip, we used samples with varying concentrations of HPV‐16 pseudoviruses and plasmids (Figure 2K,L). Standard sample preparation, as recommended by the manufacturer, served as a control experiment. Each concentration was tested in three independent trials. After extraction, the concentration of the purified nucleic acid was determined using real‐time quantitative PCR. To further underscore the importance of proper sample processing, we conducted parallel experiments in which nucleic acid extraction was done through heat lysis. The results indicated that the extraction performance of SlipChip was comparable to the standard method, with only slight variations. However, heat lysis resulted in greater sample loss. Additionally, in practical applications, the complex composition of the sample preservation fluid suggests that simple heat lysis could adversely affect downstream nucleic acid amplification.

**Figure 3:**
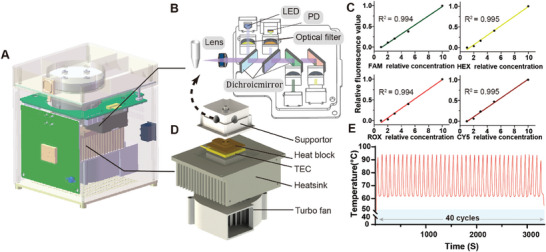
Design and characterization of the portable four‐color real‐time quantitative PCR System. A) Schematic drawing illustrating the design of the SIPOC system. B) Schematic illustration of the four‐color fluorescence detection module. C) Regression fit of the relative concentration fluorescence values for FAM, HEX, ROX, and CY5 detected by the system. D) Schematic drawing illustrating the design of the heating module. E) Representative temperature profile in the PCR chamber for 40 cycles of PCR thermal cycling.

The SIPOC system employs a portable four‐color real‐time quantitative PCR instrument for PCR amplification and detection, primarily comprising a rapid heating module and a four‐path fluorescence detection module (**Figure** **3**A). The fluorescence detection module is designed to excite and capture fluorescent light at four distinct wavelengths. It includes two structurally symmetrical black boxes, each responsible for detecting two channels of fluorescence. Each box contains a light‐gathering lens, a reflector, two LED light sources, four filters, three dichroic mirrors, and two photodiode (PD) signal acquisition modules (Figure 3B). At the front of the module, a lens focuses light to ensure strong fluorescence signal intensity. The three sets of dichroic mirrors have different coatings and are positioned at 45 or 135 angles. The principles of optical path detection are described in the supporting information. To evaluate the system's performance, we tested standard fluorescent dyes with gradient dilution, collecting and averaging 10 signal points at each concentration level. After analysis and processing, each channel displayed strong linearity, with a linear range >0.99 (Figure 3C). This high degree of linearity demonstrates the system's capability to accurately detect and quantify fluorescence signals, providing a robust platform for real‐time quantitative PCR applications. The heating module consists of a Peltier heating plate, a temperature sensor, an aluminum heat sink, and a fan (Figure 3D). The heating area is specifically designed to fit the shape of the centrifuge tube, ensuring precise and uniform heating. To facilitate fluorescence detection, small holes are cut out on both sides of the module.

To evaluate the performance of the temperature control module at room temperature (20 °C), 50 µL of deionized water and a paraffin ball were added into the reaction tubes. A type K thermocouple was inserted for temperature measurement (Figure 3E). Temperature changes in the PCR chamber were recorded over 40 thermal cycles to demonstrate the system's thermal stability and efficiency. Following hardware validation, the biological stability of the entire system was tested. The signal processing algorithm was optimized to ensure that the Ct values and their distributions from the SIPOC platform were consistent with those from the LightCycler 96 Instrument (Figure S4, Supporting Information).

**Figure 4:**
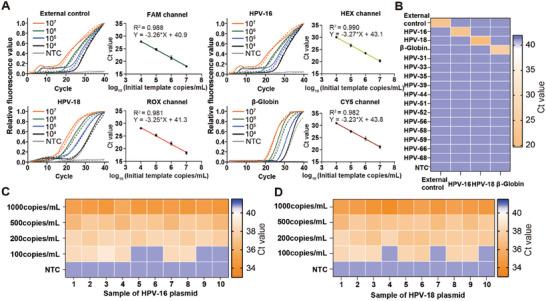
SIPOC assay evaluation. A) The amplification curves (left) and their corresponding standard curves (right) for the detection of external control, HPV‐16, HPV‐18, and β‐Globin, with concentrations ranging from 10^7^ to 10^4^ copies/mL, as well as for NTC samples. Solid and dashed lines of the same color represent parallel duplicates. The R^2^ values represent the coefficients of determination. The standard deviation (SD) is indicated by error bars for each data point (n = 3). B) Specificity characterization with HPV‐16, HPV‐18, and other high‐risk HPV viruses. There are 1000 copies in each test. C,D) Sensitivity characterization with samples containing varying concentrations of HPV‐16 and HPV‐18 plasmids.

Next, we used standard plasmids of external control, HPV‐16, HPV‐18, and the β‐Globin gene to create amplification curves and corresponding standard curves for each detection channel, with a concentration range of 10^7^–10^4^ copies/mL, as well as for NTC samples. Each PCR test for positive samples showed a strong fluorescence signal, and the standard curves exhibited a robust linear correlation (R^2^ > 0.98) (**Figure** **4**A). This high level of linearity further confirms the system's accuracy and reliability for quantitative analysis.

To evaluate the high specificity of the SlipChip‐based detection method, we tested it against 12 other common high‐risk HPV viruses and compared the results with the four targets in the detection panel (Figure 4B). The results showed that only the expected fluorescence detection channels, corresponding to their specific primer and probe sets, displayed positive signals. Additionally, gel electrophoresis experiments were performed for each detection target (Figure S6, Supporting Information), and only the target products showed bands. These findings confirm that the SlipChip method has high specificity, effectively preventing cross‐reactions and enhancing detection accuracy.

To further demonstrate the diagnostic efficacy of the SIPOC system, it is crucial to determine its limit of detection (LOD) and sensitivity. We conducted experiments using standard HPV‐16 and HPV‐18 plasmid solutions with concentrations ranging from 0 to 1000 copies/mL, using the portable PCR system for detection (Figure 4C,D). The SIPOC system consistently detected samples with 200 copies/mL and successfully identified six out of ten HPV‐16 samples and seven out of ten HPV‐18 samples at a concentration of 100 copies/mL.

**Figure 5:**
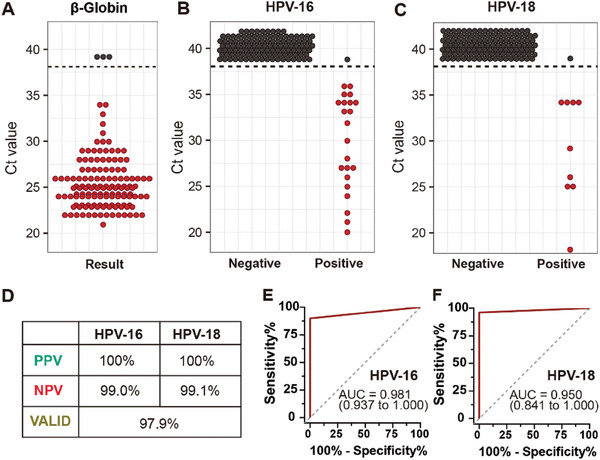
Clinical validation of the SIPOC platform. All samples were collected from patients through self‐sampling. Simultaneously, these patients follow a standardized testing protocol at the hospital to determine their current HPV infection status. Positive results are indicated by red circles, and samples with no amplification are assigned a cycle threshold (Ct) value of 39, shown as black circles. A) Results of β‐Globin detection by the SIPOC system, showing amplification for self‐sampling swabs (n = 130). B,C) Results of HPV‐16 and HPV‐18 detection by the SIPOC system, showing the amplification Ct values for self‐sampling swabs (n = 127). Horizontal dashed lines indicate the cutoff Ct values used to determine positive amplification by the SIPOC system. D) Table summarizing the PPV, NPV, and validation results for all samples. E) ROC curve for HPV‐16 identification based on amplification in all valid samples. F) ROC curve for HPV‐18 identification based on amplification in all valid samples.

To demonstrate the clinical utility of HPV screening at point‐of‐care settings, we applied the SIPOC system to analyze 130 self‐collected samples in collaboration with the International Peace Maternal and Child Health Hospital of China Welfare Society (IPMCH). The standard healthcare protocol, carried out by professionals, was used as a control. In all tests, the external control used to verify reagent performance showed positive results. (Figure S5, Supporting Information), indicating good reagent quality. Out of 130 samples, 127 tested positive for the β‐Globin gene, suggesting that 97.9% of participants successfully collected their samples, while 2.1% failed to do so (**Figure** **5**A). Notably, these three samples were also negative for the target genes when re‐tested with a standard PCR assay on the same day, confirming that the initial results were accurate and not due to reagent or assay failure. Among the valid samples, 25 tested positive for HPV‐16, 9 for HPV‐18, and 2 showed co‐infection with both HPV‐16 and HPV‐18.

Using Roche results as the reference standard, we found one clinically positive sample for HPV‐16 and one for HPV‐18 that tested negative on the SIPOC system; all other results were consistent between the SIPOC system and the Roche test (Figure 5B,C). Diagnostic sensitivity and specificity are crucial metrics for evaluating the effectiveness of cartridge assays. In this study, we assessed the diagnostic accuracy of the SIPOC system using receiver operating characteristic (ROC) curves, which illustrate the balance between sensitivity and specificity. The SIPOC system showed excellent discrimination between positive and negative samples for HPV‐16 and HPV‐18, achieving an area under the curve (AUC) of over 0.95 (p < 0.001), confirming its reliability as a diagnostic tool (Figure 5E,F).

We further evaluated the sensitivity and specificity for detecting HPV‐16 and HPV‐18 using an optimal cycle threshold of 39. The SIPOC system achieved a sensitivity of 96.2% and a specificity of 100% for HPV‐16. For HPV‐18, the sensitivity was 90.0%, while the specificity was 100% (**Table** **1**). Moreover, there was no statistically significant difference in the detection results between the SIPOC system and the clinical gold standard method for HPV‐16 (P = 1.00, exact McNemar's χ^2^ test) and HPV‐18 (P = 1.00, exact McNemar's χ^2^ test), indicating strong concordance between the methods. Additionally, the system's positive predictive value (PPV, calculated as TP/(TP + FP) × 100%) was 100%, while its negative predictive value (NPV, calculated as TN/(TN + FN) × 100%) was 99.0% for HPV‐16 and 99.1% for HPV‐18 (Figure 5D). These high predictive values further emphasize the system's accuracy and reliability in detecting HPV.

**Table 1:** Detection accuracy of HPV virus of the SIPOC system.

Target	Total samples	TP	FP	TN	FN	Sensitivity	Specificity
HPV‐16	127	25	0	101	1	96.2%	100%
HPV‐18	127	9	0	117	1	90.0%	100%

## 3 Discussion

We demonstrated that the SIPOC system, with its self‐sampling capability, can deliver results comparable to central laboratory tests while offering greater convenience, accessibility, and enhanced privacy protection. The device is designed with grooves at each stop position to ensure correct alignment, and an intuitive operation menu can instruct the user through the entire operation process (Figures S7 and S8, Supporting Information). Previous SlipChip devices were fabricated using glass substrates or polydimethylsiloxane (PDMS) for rapid prototyping and proof‐of‐concept testing; however, these methods limit the consistency and availability of the devices for routine research or clinical practice. To our knowledge, this study presents the first demonstration of the scalability of a rotational SlipChip device using injection molding with standard plastic materials, thereby achieving consistent performance and maintaining a relatively low manufacturing cost. This SIPOC system is designed to move toward the WHO's ASSURED criteria for point‐of‐care testing: affordable, sensitive, specific, user‐friendly, rapid, robust, equipment‐free, and deliverable to end users. It offers a promising solution for conducting HPV screening in developing and resource‐limited areas. With the ability to perform real‐time quantitative PCR with proper calibration, the SIPOC system has the potential to provide valuable quantitative data that could be correlated with the persistence of HPV infection and the progression stages of cervical cancer.^[^ ^41^ ^]^ Additionally, the PCR lyophilized reagents, extraction reagents, and self‐sampling kit can all be stored at room temperature for over 12 months. The versatility of the system makes it suitable for use in physicians' offices, pharmacies, and even home testing with appropriate adjustments. This flexibility could enhance both the coverage and willingness to undergo HPV testing, complementing traditional laboratory‐based methods. Therefore, the SIPOC system can be a valuable tool for the control and prevention of cervical cancer.

The system's capabilities can be further expanded to meet the requirements of various applications. Multiple instruments can be integrated and operated with a random access principle^[^ ^42^ ^]^ to increase analysis throughput. Additionally, several studies have demonstrated that PCR heating and cooling can be significantly accelerated, allowing for shorter processing times.^[^ ^43^, ^44^ ^]^ The system can also perform semi‐quantitative analysis with relative standards. To further automate the workflow, the mechanical motor and bead dispersion module can be integrated into the portable system to further reduce the manual operation steps.

The flexibility of the SIPOC system allows for enhancement with additional features. When combined with melt curve analysis^[^ ^45^ ^]^ or additional fluorescence channels,^[^ ^46^ ^]^ the system can support higher multiplex target analysis. Additionally, the SIPOC system could be compatible with various sample preparation reagents to extend its application to different sample types. By integrating other nucleic acid analysis methodologies, including loop‐mediated isothermal amplification (LAMP), recombinase polymerase amplification (RPA), nucleic acid sequence‐based amplification (NASBA), helicase‐dependent amplification (HDA), and CRISPR, the SIPOC system could serve a broader range of diagnostic purposes. With further modification and validation, the SIPOC system can become a versatile tool for point‐of‐care testing, enabling applications that go beyond HPV screening. To implement this SIPOC system in real clinical practice, further validation with additional clinical samples from cohorts in different geographical areas is needed, as the participants in this study were primarily from the Shanghai area and more sample data need to be tested. The stability of device storage and the lifespan of the instrument also require further investigation, but these aspects are beyond the scope of this paper.

## 4 Conclusion

In this work, we developed a SIPOC system with self‐sampling for point‐of‐care detection of high‐risk HPV‐16 and HPV‐18. We tested 130 samples, demonstrating that the SIPOC system can deliver performance comparable to laboratory‐based methods while offering enhanced convenience, comfort, and privacy. This portable system is easy to operate and can be scaled at relatively low cost, making it a potential tool to increase HPV screening coverage and encourage more people to undergo testing, particularly in developing and resource‐limited areas, thereby contributing to cervical cancer prevention. Additionally, it shows promise for at‐home nucleic acid testing across a broad range of diseases.

## 5 Experimental Section

### Material

All solvents and salts obtained from commercially available sources were used as received unless otherwise stated. HPV‐16 and HPV‐18 pseudoviruses were synthesized by Xinyang Leyao Biotechnology (Henan, China). Quick‐DNA/RNA Viral MagBead was purchased from Zymo Research (Irvine, CA, USA). The Taq Pro U+ Multiple Probe q‐PCR Mix Kit was ordered from Vazyme Biomedical Technology Co., Ltd. (Nanjing, China). The multiple PCR lyophilized reagents were ordered from JANZY Biotechnology Co., Ltd. (Shanghai, China). All the plasmids, primers, and probes were synthesized by Sangon Biotech (Shanghai, China). Plastic droppers were ordered from Shenghong Technology Co., Ltd. (Jiangsu, China). Magnetics were purchased from AILM Magnetic Technology Co., Ltd. (Shenzhen, China). The silicone pad was ordered from ZYM Plastic Technology Co., Ltd. (Shenzhen, China). Sealing films were ordered from KYM Material Technology Co., Ltd. (Guangdong, China). Instruction stickers were ordered from Yinshe Technology Co., Ltd. (Zhejiang, China). Paraffin wax was purchased from Sigma‐Aldrich (Sternheim, Germany). Circuit boards were ordered from Huaxia Smart Technology Co., Ltd. (Shenzhen, China). Peltier heating plates were ordered from Ferro Technology Co., Ltd. (Hangzhou, China). The turbo fan was ordered from Nidek Co., Ltd. (Kyoto, Japan). The photodiodes were purchased from Hamamatsu Hotonikusu K.K. (Hotonikusu, Japan). The thermocouple was purchased from Heraeus Holding Technology Co., Ltd. (Shanghai, China). The computing module was ordered from Giga Device Technology Co., Ltd. (Beijing, China). Wires and terminals were purchased from WAGO Ltd. (Tianjin, China).

### The Process of Self‐Sampling

The study was conducted at the International Peace Maternity and Child Health Hospital (IPMCH) in Shanghai, China, from August 9, 2023, to October 8, 2023. The trial protocol was approved by the ethics committee of the IPMCH (ethics number: GKLW 2015–45). Written informed consent was obtained from all participating patients. Participants aged 21–79 years undergoing routine cervical screening were identified and approached during their clinic visit. They were provided with an informational brochure and had a face‐to‐face discussion with a research nurse or clinician to answer any questions. Participants were given a consent form that explained the study in simple terms, including the voluntary nature of participation, the right to withdraw at any time, and confidentiality assurances. The HPV self‐sampling kit was procured from Mantacc Technology Co., Ltd. (Shenzhen, China) to improve convenience, comfort, and privacy. Among the 130 participants in this study, all successfully adhered to the self‐sampling protocol. Feedback from participants indicated a more favorable experience compared to traditional methods. The self‐sampling process entails holding the sampler with both hands, extending the swab to its full length by turning the swivel head clockwise, and inserting the sampler into the vagina until the safety stops touch the skin. Upon positioning, the sampler was rotated 3–5 times clockwise to ensure adequate contact with the vaginal epithelium before removal. Rotating clockwise was preferred due to user familiarity and provides a better user experience. After sampling, the swab was placed in the preservation buffer, shaken thoroughly, and the swab tip was broken off into the buffer. The entire process can be completed within 1 min. When analysis is required, the sample can be easily transferred to the SlipChip for preparation and subsequent testing. The ages of the 130 patients involved in this study are summarized in Table S1 (Supporting Information). Among them, three patients did not complete the self‐sampling process. The reasons for their inability to complete self‐sampling are twofold: first, their older age may have resulted in less proficiency in self‐sampling procedures; second, their test results were all negative, indicating a possible lack of subjective importance placed on HPV testing.

### The Standard Procedures for HPV Screening in Hospitals

The patient assumes the supine position on the examination table, as instructed by the physician. Using a bivalve speculum or a vaginal opener, the doctor exposes the cervix to facilitate specimen collection with a specialized HPV sampling brush. The brush was gently placed at the cervix's mouth and rotated clockwise for five turns. Subsequently, the brush was carefully removed and placed into a sampling tube labeled with the patient's identification number, which contained a specialized cell preservation solution. The brush head was then broken off into the tube, and the bottle cap was securely tightened. The sample was transported to the testing department under specified environmental conditions. Upon arrival, professionals automatically extract the HPV DNA sample using the Cobas 4800 instrument, a process involving cell digestion, decomposition, DNA release, and the absorption of magnetic glass particles for purification and elution. The resulting DNA sample undergoes detection using the Cobas 4800 HPV test system. HPV test results are typically available within 7–10 business days.

### Standard Viral RNA Extraction Process

DNA was extracted using Quick‐DNA/RNA Viral MagBead following the recommended protocol. 200 µL of sample volume was added and eluted in 75 µL of deionized water.

### Fabrication of the Injection‐Molded SlipChip

The SlipChip design was crafted using Solidworks 2016 (Dassault Systemes, MA, USA) and then brought to life through injection molding (Figure S1A,D, Supporting Information). The upper chip showcases five round wells, with three allocated for reagent storage and one designated for stress balancing. The largest well boasts a diameter of 14 mm, accommodating up to 3250 µL of liquid, while the remaining three reagent storage wells measure 7.5 mm in diameter, each with a capacity of 970 µL. Constructed from PC material, renowned for its resilience to high temperatures, robustness, and chemical inertness, the upper chip was ideal for subsequent heat sealing with aluminum foil. Its injection molding melt temperature ranges from 260 to 330 °C, with a mold temperature spanning from 70 to 130 °C, while the material particle drying temperature was set at 120 °C, requiring a minimum drying time of 4 h. The lower chip of the SlipChip system comprises a 0.5 mm thick silicone layer with a 30‐degree hardness on the top surface, complemented by an acrylic layer at the bottom acting as the adhesive agent. Crafted using AutoCAD 2010 (San Rafael, CA, USA), the solid silicone undergoes molding from controllable‐depth beads holding well, ensuring the beads remain isolated from the lower chip during experiments, thereby mitigating contamination risks. Two holes on the silicone layer facilitate the passage of the enriched nucleic acid solution post‐extraction into the nucleic acid amplification tube. Additionally, the lower chip incorporates a partially extended area beneath the holes, slightly smaller than the 0.2 mL centrifuge tube, to aid in securing both components during assembly. The centrifuge tubes house various lyophilization reagents and paraffin for sample detection, with the paraffin ball serving as a contamination deterrent. The upper and lower chips are fastened using side latches to uphold their spacing and prevent leakage during rotation.

### Characterization of the Injection‐Molded SlipChip

To assess the stability of the injection‐molded SlipChip, the repeatability of its physical parameters was initially examined using a 2.5D imaging measurement instrument (TASO Science, Suzhou, China) (Figure S1B,C, Supporting Information). Measurements of the diameter and height of 20 chips yielded coefficients of variation (CV) all below 0.5% (Figure S1E,D, Supporting Information). Subsequently, the carryover was investigated from the lysis step and the wash step into the elution step, along with the recovery rate of magnetic beads after each operational step. Food dye was spiked into the lysis buffer at a 2% volumetric ratio and into the washing buffer at a 2% volumetric ratio, and a standard curve was established (Figure S2A, Supporting Information). The measured carryover from the lysis buffer was found to be <0.1%, while the carryover from the washing buffer was 2.9% (Figure S2B, Supporting Information). The slight discrepancy in the lysis buffer value primarily stems from the systematic error of the instrument. Furthermore, the inhibitory effect of the washing buffer was assessed by introducing varying amounts into the final 50 µL amplification reagent. It was observed that washing buffer concentrations below 5% did not inhibit amplification (Figure S2C, Supporting Information), while concentrations above 5% led to a decrease in fluorescence intensity. Moreover, a standard for magnetic bead recovery was established by measuring the number of magnetic beads before and after each step using flow cytometry. The results demonstrated that the one‐step magnetic bead recovery rate exceeded 90%, while the two‐step rate exceeded 85% (Figure S3A–D, Supporting Information). This recovery rate was higher than that of standard kits, likely because the SlipChip simplifies the washing steps, reducing the loss of magnetic beads.

### The Protocol for Sample Preparation in SlipChip

In the SlipChip, lysis buffer (400 µL), washing buffer (300 µL), and eluent buffer (75 µL) were preloaded in the reagent chamber on the top chip; the bottom chip contained a magnetic bead‐holding well (2.2 µL) and an amplification tube that contained the lyophilized amplification reagent and a sealing wax ball. The lysis step involves manually shaking the SlipChip for 3 min to release the nucleic acid from the virus, then placing the device on the base station and resting for 10 s to pellet the magnetic beads into the bead‐collecting wells. During the washing step, the upper chip was rotated 60°, the SlipChip was removed from the base station, and it was manually shaken for 10 s to mix the beads with the washing buffer. After shaking, the SlipChip was placed back on the base station and left undisturbed for 10 s. For the elution step, the upper chip was rotated 60° clockwise and manually shaken for 10 s to mix the beads. The SlipChip was then returned to the base and left undisturbed for 10 s to allow the magnetic beads to pellet into the bead collecting well. To perform nucleic acid amplification, the upper chip was rotated 150° counterclockwise and manually shaken for 10 s to transfer the liquid into the tube.

### Design of the Multiple PCR Amplification Assay in the SIPOC System

Quantitative measurements of the extracted viral DNA and synthetic plasmid samples were obtained by conducting PCR using the E6 regions of the HPV‐16 and E7 regions of the HPV‐18 genome as target sequences. Due to differences in amplification efficiency and fluorescence intensity across channels, the primer and probe concentrations were optimized for each channel to ensure optimal detection performance. The reaction system had a final volume of 50 µL and included 25 µL of the Taq Pro U+ Multiple Probe q‐PCR Mix, 0.6 µL of 10 µm forward primer, 0.6 µL of 10 µm reverse primer, and 1.2 µL of 10 µm TaqMan probe for the external control channel; 1 µL of 10 µm forward primer, 1 µL of 10 µm reverse primer, and 2.2 µL of 10 µm TaqMan probe for the HPV‐16 channel; 0.5 µL of 10 µm forward primer, 0.5 µL of 10 µm reverse primer, and 1.5 µL of 10 µm TaqMan probe for the HPV‐18 channel; 0.6 µL of 10 µm forward primer, 0.6 µL of 10 µm reverse primer, and 1.2 µL of 10 µm TaqMan probe for the β‐Globin channel; and 13.5 µL of DNA templates. The RT‐PCR was performed using the following thermal cycling conditions: 40 cycles at 95 °C for 10 s and 60 °C for 30 s. The amplification process was conducted on the LightCycler 96 Instrument Real‐Time PCR System (Roche Life Science, MA, USA).

### The Principle of the Fluorescence Detection Module

In the green fluorescence channel (FAM), for example, LED light passes through a filter, is reflected by a dichroic mirror, and excites the liquid in the test tube. The resulting fluorescence from the FAM‐labeled probes was transmitted through the first dichroic mirror. The signal was then reflected at the second dichroic mirror and passed through another band‐pass filter before reaching the signal acquisition module. Given the weak fluorescence intensity in quadruple reactions, the signal acquisition module uses a photodiode (PD) with a preamplifier to capture the signal. The photodiode operated on the photoelectric effect in semiconductor materials, where incoming fluorescence photons generate electron‐hole pairs, which were converted into electrical signals, allowing for high‐sensitivity fluorescence detection. The photodiode's response was proportional to the intensity of the light within a specific wavelength range. Circuitry was designed to amplify the current and filter out noise to produce a clean electrical signal that represents the intensity of the fluorescence, aiding in the interpretation of the reaction results. In this module, four groups of excitation and emission wavelengths are adopted: the FAM channel (an excitation plate). With the advantage of the photodiode's fast light response, the detection circuit can identify four targets and acquire fluorescence signals within 2 s during the final stage of each PCR cycle extension by selectively turning the four LED light sources on and off. Furthermore, the use of dichroic mirrors and filters helps minimize crosstalk between channels.

### The Principle of the Heating Module

The PID (proportional‐integral‐derivative) temperature control algorithm was used to maintain accurate temperature regulation during PCR amplification, with a PT1000 temperature sensor embedded in the heated metal plate. This design provides precise negative feedback control during the heating and cooling stages of the PCR process. To ensure stable and sustainable thermal cycling, the system incorporates a heat dissipation module at the bottom, which features a cooling seat and a fan to remove excess heat generated during the PCR process. Additionally, the melting point of the paraffin oil used in the test tubes was ≈55 °C. When melted, it forms an insulating oil film that prevents liquid evaporation and minimizes heat loss, effectively reducing the heating time during amplification. The combination of the paraffin oil, temperature control, and cooling system ensured rapid thermal cycling.

### Characterization of PCR Products

A total of 10 µL of each PCR product was mixed with 2 µL of 6 × glycerol loading buffer (Sangon Biotech, Shanghai, China). The mixtures, along with the DL1000 DNA marker (Takara Bio, Inc., Shiga, Japan), were separated by electrophoresis on a 2% (w/v) agarose gel prepared in 1 × TAE buffer (40 mm Tris‐acetate, 1 mM EDTA, pH 8.0). Electrophoresis was conducted at a constant voltage of 110 V for ≈30 min, until the dye front had migrated an appropriate distance. The DNA bands were then visualized under UV illumination and documented using a gel documentation system.

### The Data Algorithm for Fluorescence Detection

During each cycle of the amplification reaction, four fluorescence channels collect a fluorescence signal, resulting in a total of 40 fluorescence signal values. Typically, the baseline in conventional PCR amplification reactions was set as the average fluorescence signal of the first 15 cycles, while the fluorescence threshold was set as ten times the standard deviation of the fluorescence signal from cycles 3 to 15. However, setting the threshold may be influenced by atypical data generated during real‐time quantitative PCR experiments. To address the limitations of traditional methods, the data processing algorithm was optimized by introducing a dynamic threshold adjustment based on the difference in fluorescence curve amplitudes, thereby achieving adaptive threshold setting. Initially, these data were normalized, followed by fitting the normalized data using an S‐shaped curve. Subsequently, the threshold was drawn based on the standard deviation of the blank signal and preceding data, and the algorithm was further optimized based on the results obtained from the standard PCR instrument. To validate the feasibility of this algorithm, the study simultaneously amplified viral DNA plasmids using both the SIPOC platform and the LightCycler 96 instrument, resulting in consistent results between the two platforms.

### Statistical Analysis

All clinical data were analyzed using GraphPad Prism version 8.0.2 and SPSS version 26.0. ROC curves were generated to evaluate the diagnostic performance, with calculations of sensitivity, specificity, and the AUC. To assess the agreement between the SlipChip technique and the standard method, an exact McNemar's χ^2^ test was performed using SPSS version 26.0. A p‐value < 0.05 was considered statistically significant for all tests.

## Conflict of Interest

The authors declare no conflict of interest.

## Author Contributions

J.Z. and Z.D. contributed equally to this work. All authors contributed to the writing of this manuscript. J.Z. contributed to the methodology, investigation, writing of the original draft, review and editing, and visualization. Z.D. assisted with sample provision and collection, investigation, and writing of the original draft and review and editing. L.X. was involved in the methodology, investigation, writing of the original draft, review and editing, and visualization. X.H. also contributed to the methodology, investigation, writing of the original draft, and visualization. Z.S. participated in the investigation and writing of the original draft. W.C. focused on writing the original draft and data analysis, while J.Z. handled writing the review and editing and data analysis. D.L. contributed to conceptualization, sample provision and collection, as well as writing review and editing. Lastly, F.S. played a key role in conceptualization, methodology, writing of the original draft, review and editing, and visualization.

## Supporting information



Supporting Information

## Data Availability

The data that support the findings of this study are available on request from the corresponding author. The data are not publicly available due to privacy or ethical restrictions.
