# Supplementary material for: An Injection Molded SlipChip with Self‐Sampling for Integrated Point‐of‐Care Testing of Human Papilloma Virus
Source: Adv Sci (Weinh). 2024 Sep 25;11(43):2406367. doi: 10.1002/advs.202406367 (PMC11578293; doi:10.1002/advs.202406367)
Supplement: Supplementary file 1 — Supporting Information [file ADVS-11-2406367-s001.docx]

Supporting Information

**An Injection Molded SlipChip with Self-sampling for Integrated Point-of-Care Testing of Human Papilloma Virus**

*Jiajie Zhang^1^, Zhangli Dong^1^, Lei Xu, Xu Han, Zheyi Sheng, Weiyu Chen, Jiayi Zheng, Dongmei Lai*, Feng Shen**

1 J. Z. and Z. D. contributed equally.

This file includes

Tables S1–S3

Figure S1–S8

**Supplementary tables and figures**

**Table S1. Self-sampling completion rate of different age range**

| Age range | Numbers | Self-sampling completion rate |
| --- | --- | --- |
| 20-29 | 17 | 100% |
| 30-39 | 35 | 100% |
| 40-49 | 27 | 100% |
| 50-59 | 19 | 94.7% |
| 60-69 | 24 | 95.8% |
| 70-79 | 8 | 87.5% |
| Total | 130 | 97.7% |

**Table S2. The cost of the SIPOC system**

| System | Name（amount） | Function | Total Cost(＄) |
| --- | --- | --- | --- |
| electronics | MCU | microcontroller | 9.50 |
| electronics | SMD IC | signal conversion | 3.50 |
| electronics | wires＆ pins | connection | 2.30 |
| electronics | SMD capacitors | power stabilizing | 1.11 |
| electronics | switch | control the base station | 0.83 |
| magnet | magnet | control the magnetic beads | 0.53 |
| thermal | chip resistors | temperature control | 0.35 |
| thermal | heat sink | heat transfer | 7.00 |
| thermal | fan | cooling | 8.00 |
| thermal | Peltier heating plate | heater | 23.00 |
| thermal | thermocouple | temperature sensing | 4.00 |
| thermal | insulate plate | insure the contact of heating | 0.82 |
| optical | dichroic mirror (6) | reflection and refraction of light | 56.50 |
| optical | filter (4) | retention of required wavelengths | 21.30 |
| optical | photodiode (4) | fluorescence signal collection | 53.40 |
| optical | LED (4) | light source | 1.20 |
| optical | reflector (2) | light path control | 13.50 |
| optical | lens (2) | light focusing | 3.80 |
| structure | ABS structure | body shape | 15.70 |
| structure | screws | assembly | 0.60 |
| others |  |  | 29.70 |
|  |  | **Total cost of goods** | 256.64 |

**Table S3. Sequences of primers for SIPOC assay**

| Name | Description | Sequence (5′ to 3′) | Region | Source |
| --- | --- | --- | --- | --- |
| External control | Forward primer | GGAGCAAGAGCCCGGAGT |  |  |
|  | Reverse primer | CCAATAAAGTGGCCACCACTT |  |  |
|  | Probe | FAM-CGTGATAAGGGAACCTCGAGTGCCT-BHQ1 |  |  |
| HPV-16 | Forward primer | AGGAGGAGGATGAAATAGATGGTC | E6 | Zai Y, et al ^[1]^. |
|  | Reverse primer | CCCATTAACAGGTCTTCCAAAGTAC |  |  |
|  | Probe | HEX-CCTTTTGTTGCAAGTGTGACTCTACGC-BHQ2 |  |  |
| HPV-18 | Forward primer | CGCGCTTTGAGGATCCAAC | E7 | Zai Y, et al ^[1]^. |
|  | Reverse primer | CACAGGTTATTTCTATGTCTTGCAGT |  |  |
|  | Probe | ROX-CGGCGACCCTACAAGCTACCTGATCT-BHQ2 |  |  |
| β-Globin | Forward primer | GCTTACATTTGCTTCTGACAC |  | Zai Y, et al ^[1]^. |
|  | Reverse primer | AGTAACGGCAGACTTCTCC |  |  |
|  | Probe | CY5-TCACTAGCAACCTCAAACAGACACCAT-BHQ3 |  |  |


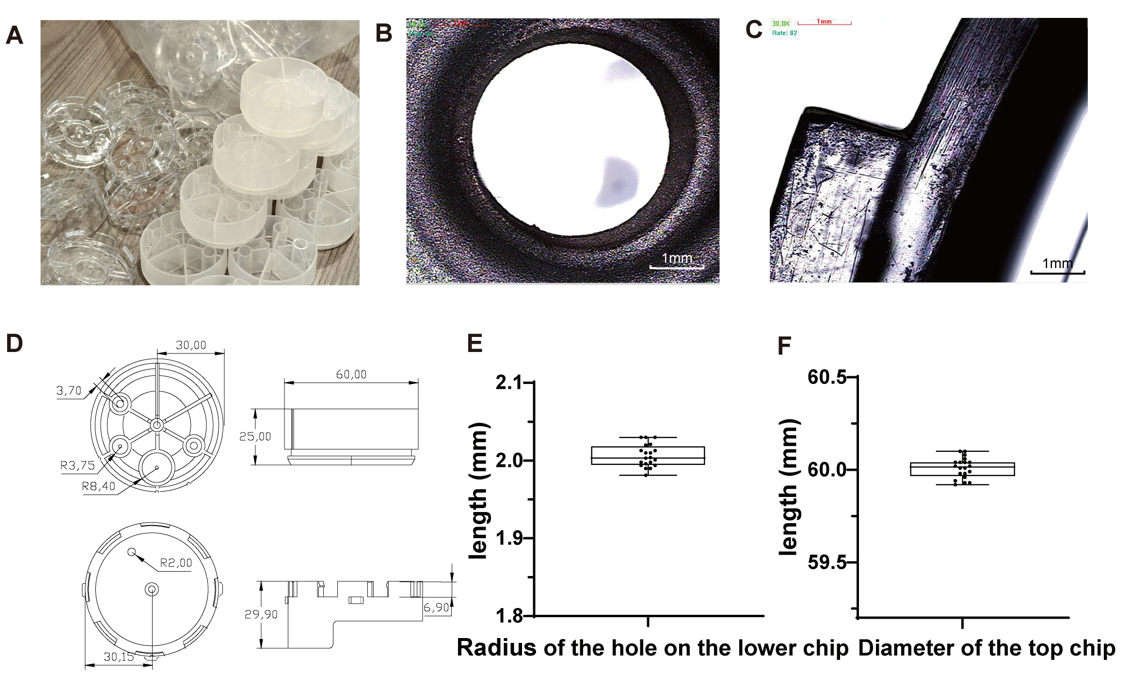


**Figure S1.** The photograph and the characterization of the injection-molded SlipChip Dimensional drawing of the SlipChip. (A) Photograph of the injection-molded SlipChip. (B-C) Photograph of the injection-molded SlipChip under the 2.5D imaging measurement instrument. (D) Dimensional drawing of the SlipChip (E) Box and whisker charts for the length of the diameter of the hole in the SlipChip (n = 20) (F) Box and whisker charts for the length of the diameter of the top chip (n = 20)


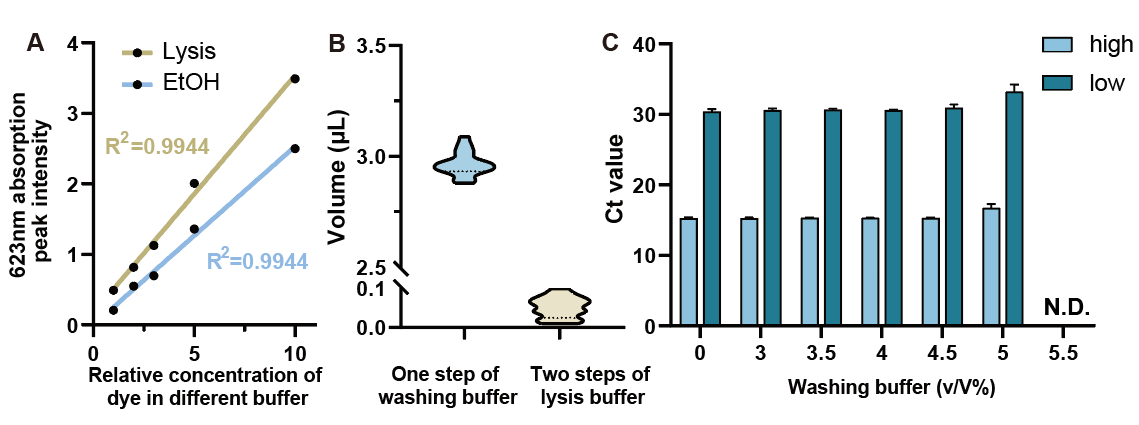


**Figure S2.** The characterization of carryover. (A) Regression fit of the relative concentration of dye in lysis and EtOH. (B) Violin plots for the carryover volume for one step of washing buffer and two steps of lysis buffer (n = 10). (C) Inhibitory effect caused by the carryover. There are 10^6^ copies of HPV-16 DNA in each high test and 1000 copies of HPV-16 DNA in each low test. Different proportions of washing buffer in the reaction solution: the sample shows no signal when the volume fraction of washing buffer is greater than 5%. The error bars represent the standard deviations (n = 3).


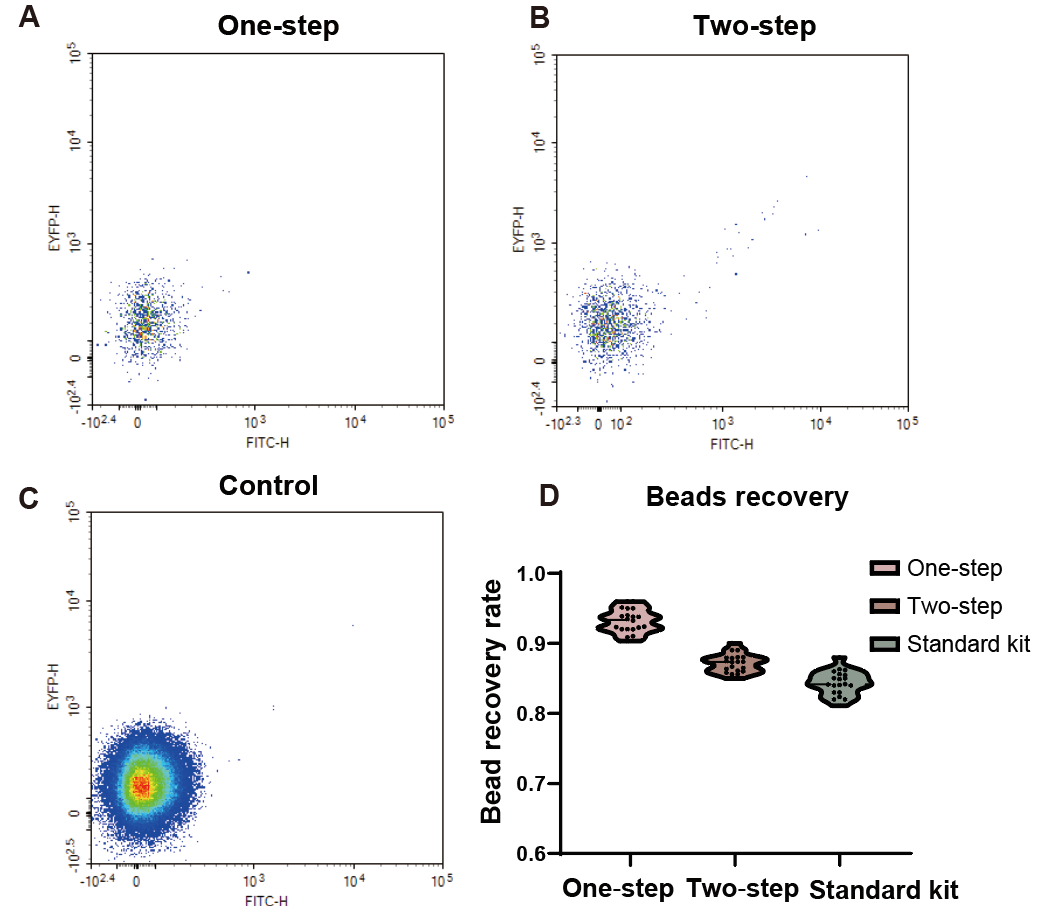


**Figure S3.** The characterization of beads recovery rate. (A-C) Measurement of bead quantity using flow cytometry for one-step operation, two-step operation, and 5 μL bead suspension, with the tested liquids diluted in the same proportion. (D) Violin plots for the beads recovery rate at one-step, two-step, and the standard kit protocol (n = 20).


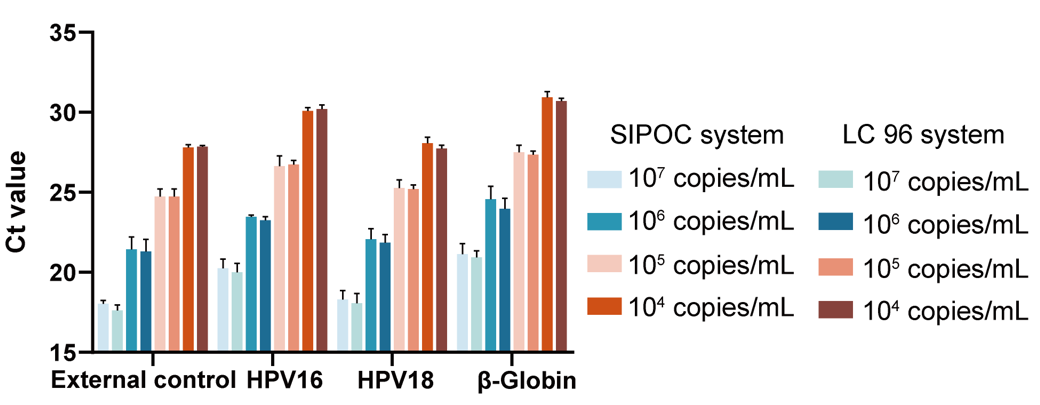


**Figure S4.** Comparison of the Ct values using the SIPOC system (blue) and the gold standard instrument LightCycler 96 (red), across the four detection channels. The error bars represent the standard deviations (n = 3).


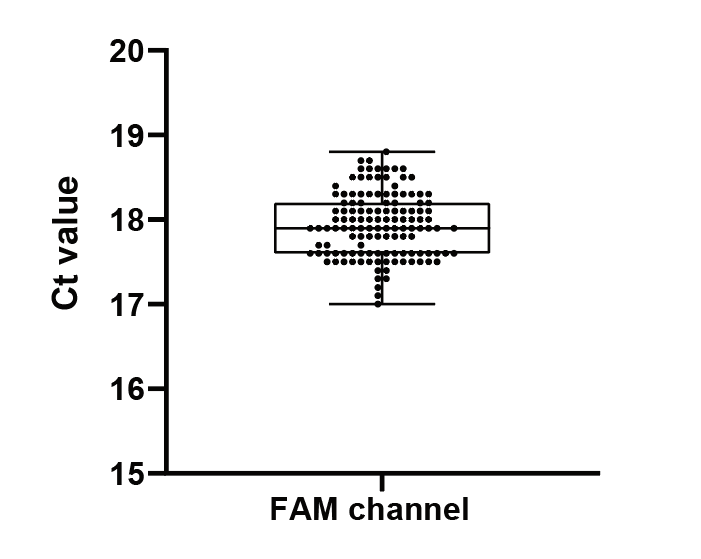


**Figure S5.** The data from the external control of the 130 samples. The Ct value of the external channel (FAM).


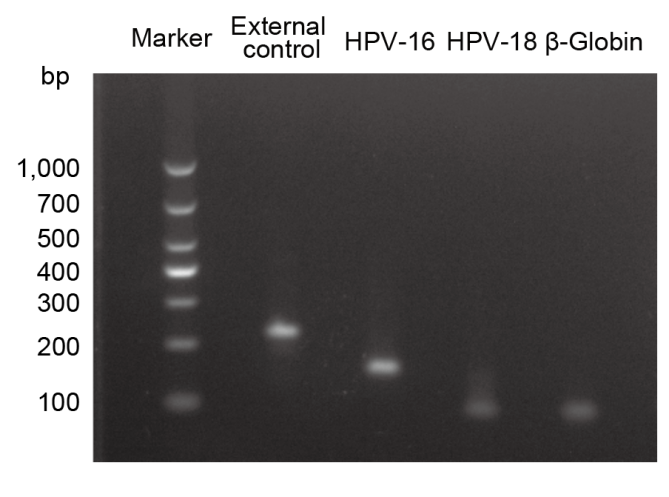


**Figure S6.** Analysis by gel electrophoresis of PCR reaction product from plasmids containing the external control, HPV-16, HPV-18, and the β-Globin gene. Each lane shows a distinct single amplification product: 224 bp for the external control, 157 bp for HPV-16, 93 bp for HPV-18, and 93 bp for the β-Globin gene.


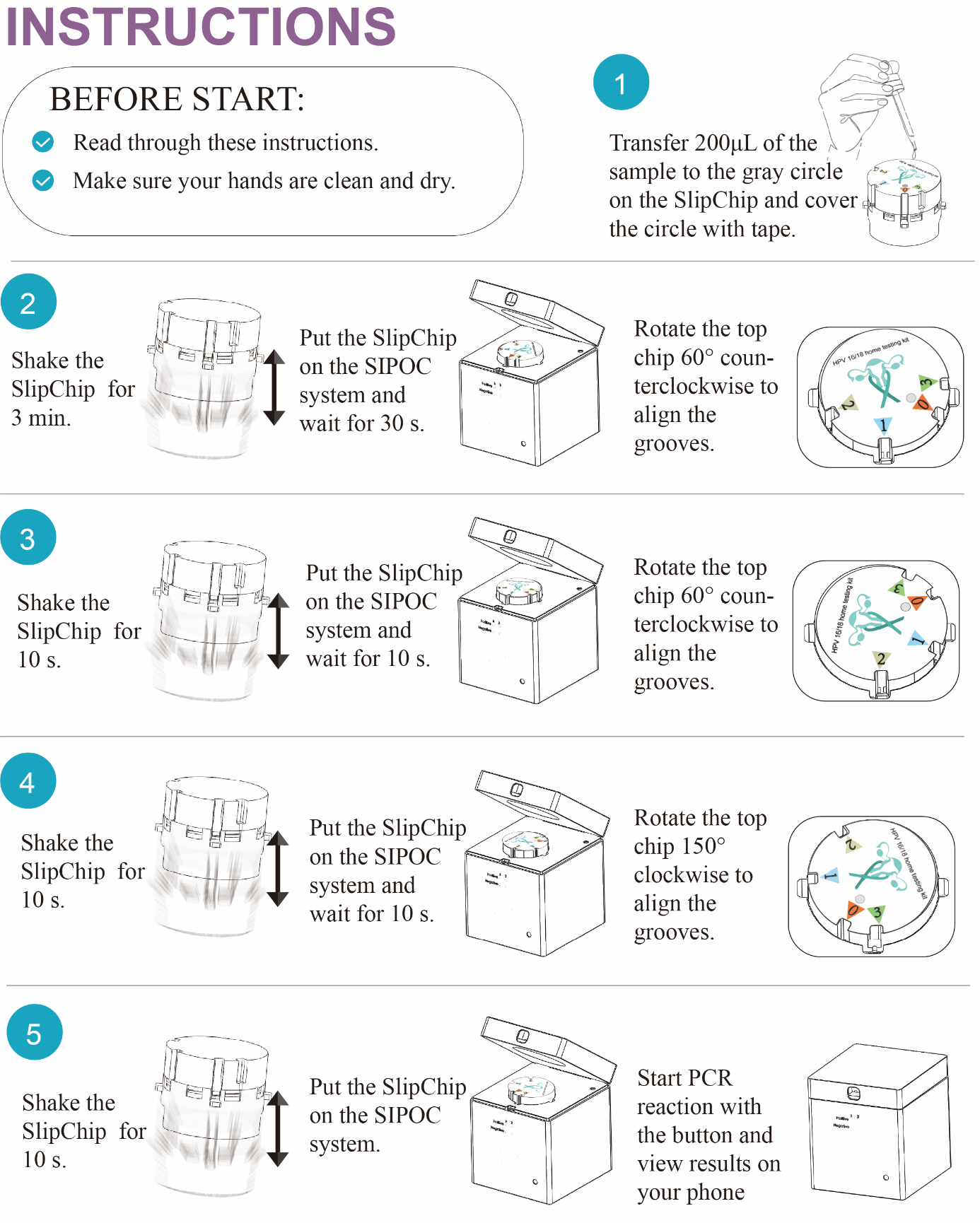


**Figure S7.** The operation instruction of SIPOC.


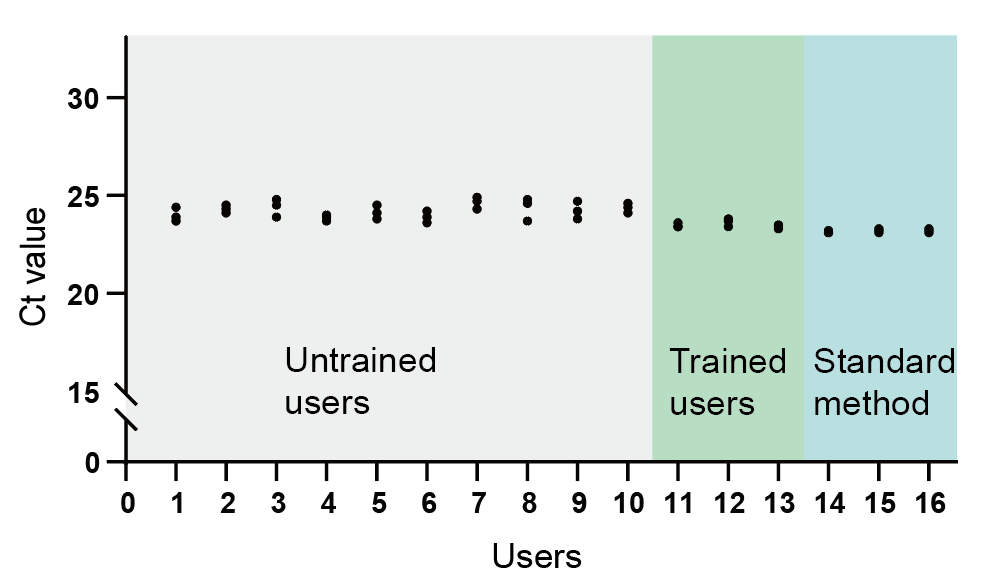


**Figure S8.** Comparison of the SlipChip method for sample preparation between untrained and trained users. Ten untrained users and three trained users each performed three nucleic acid extractions using an HPV-16 plasmid concentration of 10^6^ copies. Solid circles represent the Ct values for each sample. The gray area indicates the results from untrained users, the green area represents the results from trained users, and the blue area represents the standard method.

**Reference:**

[1]. Zai, Y.; Min, C.; Wang, Z.; Ding, Y.; Zhao, H.; Su, E.; He, N., *Lab Chip* 2022, **22**, 3436.
